# Bacteria-Mediated Modulatory Strategies for Colorectal Cancer Treatment

**DOI:** 10.3390/biomedicines10040832

**Published:** 2022-04-01

**Authors:** Anna-Lena Mueller, Aranka Brockmueller, Niusha Fahimi, Tahere Ghotbi, Sara Hashemi, Sadaf Sadri, Negar Khorshidi, Ajaikumar B. Kunnumakkara, Mehdi Shakibaei

**Affiliations:** 1 Musculoskeletal Research Group and Tumor Biology, Chair of Vegetative Anatomy, Institute of Anatomy, Faculty of Medicine, Ludwig-Maximilian-University Munich, 80336 Munich, Germany; a.mueller@med.uni-muenchen.de (A.-L.M.); aranka.brockmueller@med.uni-muenchen.de (A.B.); 2 Faculty of Pharmacy, Comenius University, 83232 Bratislava, Slovakia; niyoosha.fahimi@yahoo.com; 3 Department of Nursing, Shiraz University of Medical Sciences, Shiraz 7134814336, Iran; ghotbit@sums.ac.ir; 4 Central Tehran Branch, Islamic Azad University, Tehran 1955847881, Iran; shashemi336@gmail.com; 5 Department of Microbiology, University of Mazandaran, Babolsar 4741613534, Iran; dr.sf1376@gmail.com; 6 Department of Medicinal Chemistry, Medical Sciences Branch, Islamic Azad University, Tehran 1913674711, Iran; n.khorshidi73@yahoo.com; 7 Cancer Biology Laboratory and DBT-AIST International Center for Translational and Environmental Research (DAICENTER), Department of Biosciences and Bioengineering, Indian Institute of Technology (IIT) Guwahati, Guwahati 781039, India; kunnumakkara@iitg.ac.in

**Keywords:** colorectal cancer, biotherapeutical toxins, bacteriocins, bacterial peptides, bacteriotherapy, microbiota

## Abstract

Colorectal cancer (CRC) is one of the most common tumors worldwide, with a higher rate of distant metastases than other malignancies and with regular occurrence of drug resistance. Therefore, scientists are forced to further develop novel and innovative therapeutic treatment strategies, whereby it has been discovered microorganisms, albeit linked to CRC pathogenesis, are able to act as highly selective CRC treatment agents. Consequently, researchers are increasingly focusing on bacteriotherapy as a novel therapeutic strategy with less or no side effects compared to standard cancer treatment methods. With multiple successful trials making use of various bacteria-associated mechanisms, bacteriotherapy in cancer treatment is on its way to become a promising tool in CRC targeting therapy. In this study, we describe the anti-cancer effects of bacterial therapy focusing on the treatment of CRC as well as diverse modulatory mechanisms and techniques that bacteriotherapy offers such as bacterial-related biotherapeutics including peptides, toxins, bacteriocins or the use of bacterial carriers and underlying molecular processes to target colorectal tumors.

## 1. Introduction

Colorectal cancer (CRC) is globally among the most common causes of cancer-related death, whereby 50% of patients who are not showing metastasis when diagnosed, will develop metastases with the progressing course of the cancer disease [1,2,3,4], with the most common sites being liver and lungs [3]. CRC is known to be affected by environmental and lifestyle factors including poor diet, physical inactivity and a sedentary lifestyle [5,6]. The pathogenesis of CRC is characterized by multiple factors contributing to the disease, such as genetic mutations and epigenetic alterations as well as building of and interaction with the tumor microenvironment (TME) that promotes further tumor progression and metastasis [7,8,9]. Hereby, chronic inflammation, known as a risk factor for CRC development, plays a pivotal role, since diverse pro-inflammatory mediators such as cytokines, chemokines, carcinogens, chemotherapeutic substances or radiation, have been demonstrated to further stimulate inflammatory pathways (e.g., nuclear factor ‘kappa-light-chain-enhancer’ of activated B-cells, NF-κB), leading to tumor cell proliferation and invasion [10,11,12,13]. Moreover, it has been frequently shown that inflammatory bowel diseases and bowel-linked inflammation are not uncommonly associated with CRC tumor progression, highlighting the role of the gut’s inflammation-protective capability [14].

The gut, as a tissue hosting approximately 3 × 10^13^ colonic bacteria, is assumed to be responsible for the majority of known microbial immunomodulatory effects and immunity in the intestinal tract as well as for metabolism and inflammation and even shows cancer-protective properties [15,16,17,18,19]. This effect has already been discovered in the late 1800 s by William B. Coley, who was able to demonstrate tumor reduction and extended survival of CRC patients by using a mix of bacterial species *Serratia marcescens* and *Streptococcus pyogenes* for the treatment of sarcomas [20].

Today, bacterial therapy has been rediscovered as a potential treatment strategy for CRC [18,21,22,23], especially because tumor cells are capable of genetic mutations, and are thus able to escape from immune monitoring and can even develop resistance to standard immunotherapies. Moreover, current anti-tumor therapeutics are associated with high toxicity to normal cells, finally leading to severe side effects in patients, thus current cancer treatment is frequently exposed to a number of drawbacks [16,24,25]. Therefore, using bacteria strains possessing anti-cancer properties represents a promising strategy as preventative, concomitant or alternative treatment of CRC [18,20,21,22,23].

In particular, bacterial peptides, including toxins, show characteristics such as low molecular weight and hydrophobicity, facilitating their entry into tumor tissue, where they can unfold their anti-cancer effects [26]. Furthermore, taking advantage of the fact that some bacteria show tumor targeting specificity, they have also been used as carriers for anti-tumor agents and even for tumor and metastases detection in previous studies [27,28,29,30,31]. Besides, using bacteria as probiotics has been presented as another application strategy in the treatment of CRC and its prevention, showing direct effects by suppressing carcinogens and stimulating immune modulation [32,33,34,35]. This demonstrates again the various methods of bacterial application in CRC treatment approaches and its anti-cancer potential on various levels.

In this review, we will present a wide range of recently demonstrated treatment methods using bacteria for cancer therapy, whereby different bacterial mechanisms and their properties for treatment application will be described with a focus on CRC.

## 2. Bacteriotherapy in CRC Treatment

In recent decades, the mortality rate of various cancers has remarkably increased, forcing scientists to further develop novel and innovative therapeutic treatment strategies, whereby bacterial therapy has been shown to be a very promising one [36], especially regarding CRC, which ranks among the most prevalent life-threatening types of cancer, bacteriotherapy provides an attractive novel and cost-efficacious treatment approach. Accordingly, research has shown that the microbiome of patients suffering from CRC has a significantly different composition than that of healthy individuals [37,38]. Moreover, it is known that pathogenic bacteria and microorganisms can greatly contribute to the development of CRC, but on the other hand, others were found to act as effective therapeutic agents with less or even no side effects compared to standard cancer treatment [20,39]. Based on this background further research on bacteria’s role in the treatment of patients suffering from CRC appears to be very promising.

### 2.1. Mechanisms Used in Bacteriotherapy in CRC

There have been several mechanisms of bacteria described to date that researchers are taking advantage of in the treatment of CRC via bacteriotherapy, such as formation of pores in the cell membrane, inhibition of metastasis, tumor necrosis or apoptosis [20,40].

**Figure 1:**
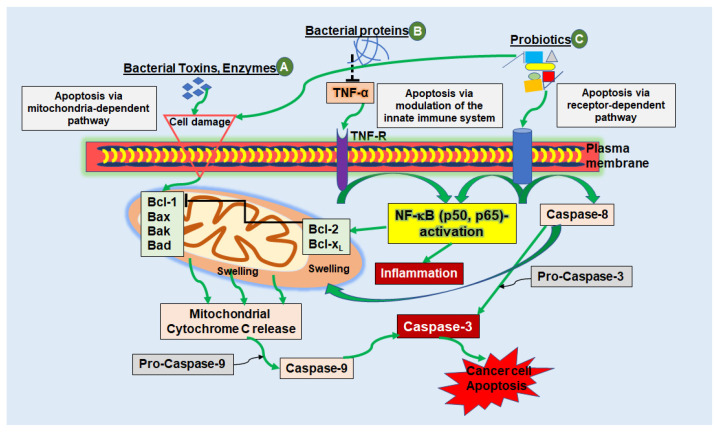
Schematic diagram showing the mechanisms of apoptosis triggered by bacterial peptides in cancer cells. (**A**) Bacterial toxins, secreted by various bacterial strains can cause apoptosis via the mitochondria-dependent pathway by causing cell injury, for example, by cell membrane pore formation. Induction of the intrinsic pathway leads to activation of pro-apoptotic proteins (Bcl-1, Bad, Bax, Bak), which in turn stimulates the release of cytochrome c molecules from the mitochondrial intermembrane space into the cytosol. Cytochrome c, together with Caspase-9 forms a complex called the “apoptosome”, finally stimulating executioner caspases (e.g., Caspase-3) leading to cancer cell apoptosis. (**B**) Bacterial proteins and peptides can have a modulatory impact on cytokines such as TNF-α, resulting in activation or blockage of NF-κB. With suppression of NF-κB, which stimulates anti-apoptotic proteins Bcl-2 and Bcl-xL, which in turn regulates apoptosis by blocking cytochrome c release, pro-apoptotic Bax and Bak-proteins remain stimulated and apoptosis is induced. (**C**) Besides stimulating the intrinsic pathway of apoptosis, probiotics are capable of apoptosis induction through stimulation of the extrinsic receptor-dependent pathway. Here, so called cell death receptors, such as TNF-R, bind to natural ligands, whereby initiator Caspase-8 and -10 are activated to cleave further downstream caspases, such as Caspase-3, which in turn induces cell apoptosis [41,42,43,44,45].

In particular, apoptosis has been known as a key goal in cancer treatment for several decades in order to eradicate tumor tissue that is characterized by a loss of balance between cell proliferation and death [41,42]. The term apoptosis or programmed cell death, describes a very complex process, which includes various pathways and mechanisms (Figure 1) [42,43]. In general, it is distinguished between the intrinsic (mitochondrial-dependent) pathway and the extrinsic (receptor-dependent) pathway, both finally leading to Caspase activity and apoptosis. Programmed cell death, stimulated by the intrinsic pathway, is characterized by cytochrome c release from pro-apoptotic proteins (B-cell lymphoma 1-Bcl-1, Bcl-2-associated X protein-Bax, Bcl-2-Antagonist of Cell Death-Bad and BCL2 Antagonist/Killer 1-Bak)-stimulated mitochondria from the intermembrane space into the cytosol, subsequently forming the apoptosome complex together with Caspase-9, eventually leading to apoptosis. The extrinsic pathway on the other hand is stimulated by cell membrane death receptors such as Tumor necrosis factor receptor (TNF-R) binding to natural ligands, whereby initiator Caspase-8 is induced, which promotes cleavage of further downstream caspases, finally inducing apoptosis [41,42,44,45]. Moreover, receptor-ligand binding induces several cellular responses, including the activation of NF-κB, which can activate pro-apoptotic proteins depending on the cellular context [44,45]. However, the two main apoptotic pathways must not be considered separately, since previous research showed that they are linked with each other and metabolites of one pathway can have an impact on the other [45].

Altogether, to make use of these mechanisms such as bacteria-induced apoptosis and metastasis suppression and to establish efficient therapy methods within using bacteria, it is important to meet several framework conditions such as maximum cytotoxicity against cancer cells with minimum cytotoxicity towards intact cell tissue and the ability to selectively attack carcinomas [20,40].

In the following section, different mechanisms that have been made use of in bacterial cancer therapy are described with a particular focus on the main topics of interest related to CRC.

#### 2.1.1. Bacteriotherapy for Modulating Innate Immunity

Our immune system plays a crucial role in the protection and defense against cancer development [46]. Here, it is of great importance to understand the duality of the immune system composed of a defensive and a reparative mode. While the defensive mode promotes the production of immune cells, the reparative mode stimulates the secretion of immune suppressive cytokines, and growth factors among others, thus facilitating cell invasion. Bacteria can interact either as pathogens or as compounds of the “normal” flora, whereby pathogenic interaction of bacteria triggers immune system activity in several ways, described in the following section [36,47,48].

*Activation of Inflammasome Pathways*

One mechanism the immune system is activated by, is represented by the stimulation of inflammasome pathways, triggered by bacteria. Previous investigation on the ∆ppGpp *Salmonella typhimurium* strain, which is defective in the synthesis of ppGpp (regulates virulence gene expression), demonstrated its tumor-targeting activity in CRC mouse models by activating inflammasome pathways, leading to suppression of tumor cell released signals and significantly increased levels of the pro-inflammatory cytokine IL-1ß in the tumors, resulting in tumor growth inhibition [38,49]. Another mechanism stimulating the inflammasome pathway besides direct activation through the strain of ∆ppGpp *Salmonella typhimurium* revealed, was demonstrated by the secretion of ATP from damaged cancer cells attacked by ∆ppGpp *Salmonella typhimurium*, leading to NLRP3 inflammasome activation in macrophages [38]. Moreover, in another study of ∆ppGpp *Salmonella typhimurium*, researchers found evidence that the bacterial strain shows tumor-targeting ability and that primary as well as even metastatic CRC could be suppressed in mice [50]. Based on this background, the mechanism of activating the inflammasome by bacterial strains can be considered as an efficient therapeutic strategy to make use of in bacteriotherapy in CRC treatment.

*Activation of Anti-Tumor T-cell Responses*

Another mechanism that has been demonstrated to inhibit tumor development refers to anti-tumor effectors T-cell responses. Here, *Escherichia coli*, an anaerobic bacteria strain, was found to be indirectly involved in CRC clearance via activating as the host’s defense mechanism, leading to T-lymphocyte production. These, in turn, greatly contribute to tumor-protective activity by CD8^+^ and CD4^+^ T-cells acting as major effectors in the immunological response against tumors as previously shown in CRC mouse models [38]. Importantly, it was proven via depletion experiments that CD8^+^ T-cells were the only effectors during the induction phase, thus responsible for tumor clearance, while both, CD8^+^ and CD4^+^, were involved in the memory phase. In addition, the anti-tumor T-cell effectors (CD4^+^ and CD8^+^) were found to suppress newly set tumors, whereby CD8^+^ T-cells were even able to destroy already established CRC [38]. Moreover, in another study CD4^+^ and CD25^+^ regulatory T-cells have been shown to reduce the risk of colon cancer, underlining the potential of bacteria triggered T-cell responses as a novel CRC treatment approach [51].

*Activation of Cytokine-Triggered Tumor Necrosis*

Tumor-colonizing bacteria describe specific anaerobic bacteria species that invade and are able to grow in solid tumors because damaged circulation and necrosis found in tumors present necessary condition for anaerobic bacteria to grow and replicate [52,53,54,55,56]. In a study of Leschner et al. the administration of anaerobic *Salmonella enterica serovar Typhimurium* to cancer mouse models showed that through hemorrhaging a remarkable increase in bacterial flushing into the solid tumor and subsequent necrosis could be detected, which is associated with TNF-α secretion and its capability to destroy vascular endothelial cells. Based on this background, activation of the innate immune system and subsequent TNF-α secretion can play a pivotal role in tumor necrosis [56]. In addition to that, in another study, application of *Salmonella typhimurium*, *Shigella flexneri* and *Escherichia coli* to cancer-bearing mice lead to their accumulation and proliferation within the solid tumors, especially in necrotic regions rather than viable tumor cells, whereby it has been shown that the number of neutrophilic granulocytes as an active part of the innate immune system enhancing TNF-α secretion is proportional to the area of necrosis. Moreover, bacteria were also then able to migrate into vital tumors, finally resulting in an increased size of necrosis, highlighting the anti-tumor properties of TNF-α by further enhancing and modulating the immune response at least in this kind of cellular context [57]. These findings also further demonstrate the potential of bacteria-mediated modulation of the innate immune system as a novel treatment strategy in CRC therapy.

#### 2.1.2. Bacterial Peptides

Peptides acting as anti-tumor agents, including several bacterial peptides, are mainly characterized by having low molecular weight and hydrophobicity. These properties seem to be of great importance for the peptides to migrate into the tumor, where cells partly exhibit an altered surface compared to normal cells. Within the tumor, bacterial peptides can unfold their anti-cancer and immune-modulating activity in different ways dependent on their characteristics [26].

*A: Bacterial Toxins*

The use of bacterial peptides as bacterial toxins demonstrates another CRC treatment approach. Bacterial toxins are substances and metabolites secreted by bacteria that have been shown to suppress tumor growth in numerous studies [58]. They are synthesized by almost all bacterial species and have already been identified for a large number of bacterial strains. Under physiological conditions, bacteriocins help bacteria to protect themselves from competing microorganisms by killing them [59,60]. So far, two types of bacterial anti-cancer toxins have been identified: toxins that conjugate to surface antigens of malignant cells and secondly toxins that conjugate to ligands of cancer cells [61]. Since CRC cells present a great number of tumor-specific antigens on their surface, mainly functioning as receptors, bacterial toxins represent a powerful tool to specifically bind them [60]. In the following, we will present some of these bacterial toxins that have been demonstrated to be effective against CRC (Table 1).

*Enterotoxin*

Enterotoxin (CPE) for example, which is produced by *C. perfringens*, a gram-positive anaerobic bacterium, represents one of the most effective bacterial toxins used in CRC therapy by binding to Claudin-3 and -4 surface receptors that are prevalent in malignant cells, which finally leads to cellular osmotic balance breakdown and cancer cell lysis [62,63]. In addition, Pahle et al. found an optimized CPE expressing vector that targets Claudin-3 and Claudin-4 expression in SW480, HCT-116, SW620, Caco-2, HT-29 and PDX colon cancer cells, demonstrating CPE as a gene transfer system that could be used as a therapeutic agent in CRC treatment directed against Claudin-3 and -4 that causes fast tumor cell death, as shown in vitro and in vivo [62,63]. Moreover, several other bacteriocins showing anti-cancer properties have been found, such as Diphteria toxin (DT), Nisin, Colicin, Microcin and Pediocin [64].

*Diphteria Toxin*

Diphteria toxin, consisting of two subunits A and B, is released by gram-positive Corynebacterium diphtheria bacteria. Via subunit B, the bacterium is able to bind to cancer cells, while the catalytically active subunit A is able to block the protein synthesis via ADP-ribosylation of cytoplasmic elongation factor 2 (EF-2), finally resulting in cancer cell death [65,66]. A non-toxic attenuated form of DT (CRM197) has been demonstrated to bind to HBEGF (heparin-binding epidermal growth factor) and to suppress cancer proliferation and angiogenesis while inducing apoptosis in vivo. These anti-cancer effects of DT have also been found in the treatment of colon cancer cell lines SW480, SW620, HCT116, CaCo-2 and HT-29 [67,68].

*Nisin*

Nisin, a polycyclic peptide secreted by the bacterial strain *Lactococcus lactis*, has shown a significant cytotoxic effect on Caco-2 and HT-29 CRC cell types, whereby hole formation in the cell membrane of target cancer cells was promoted, which finally lead to cytoplasmic membrane depolarization and apoptosis [69]. Furthermore, Nisin has also been demonstrated to prevent metastatic gene expression of MMP-2, MMP-9, cytolethal distending toxins and the cycle inhibiting factor in CRC cell lines, namely LS-180, HT-29, SW480 and Caco-2, further supporting Nisin’s role as an anti-cancer agent [70,71]. The application of Nisin, combined with conventional therapy, has already been shown to help reduce the therapeutic doses of various anti-cancer medications by increasing their cytotoxicity [71].

*Colicin*

The aforementioned bacteriocin colicin is produced by *Enterobacteriaceae* such as *E. coli*. It is assumed that colicins take cytotoxic action on various malignant cells through membrane hole formation, a non-specified DNase and RNase activity and inhibition of the murein synthesis [72]. In previous research, the inhibitory effect of Colicin E1, E3, A, U on growth of HT-29 CRC cells among other human cancer cell lines was demonstrated [73], whereby HT-29 cells reacted insensitive to Colicin E1, while Colicin A treatment provoked the highest cytotoxicity against HT-29 cells [74].

*Microcin*

According to studies that were carried out to evaluate the impact of Microcin E492, secreted by gram-negative *Klebsiella pneumonia*, on different cancer cell lines such as HeLa, Jurkat, RJ2.25 and also CRC cells, its anti-tumor activity could be demonstrated. Hereby the main mode of action of Microcin E492 included pore formation in cancer cell membranes, finally leading to apoptosis by binding to Toll-like-receptor (TLR) 4 [75,76]. Interestingly, inhibition of normal cells such as bone marrow cells, splenocytes, KG-1 or human tonsil cells has not been observed, demonstrating bacteria to selectively target malignant cells [75,77].

*Pediocin*

Another type of bacteriocin that has been shown to have lethal effects on CRC cells (HT-29 and DLD-1) probably due to its hydrophobic nature, is the bacterial toxin pediocin, which is derived from *Pediococcus acidilactici* (K2a2–3), a gram-positive bacterial strain that is able to grow in a wide range of pH, temperature and osmotic pressure. These treatments enable them to enter and grow in the intestinal tract [78]. The specific mechanism underlying pediocin’s cytotoxic effect on cancer cells is not fully understood yet, but application of sequence alignment has demonstrated great homology between Pediocin PA-1 and Microcin E492. Since the latter has been studied in more detail and is known to interact with TLR4 for induction of apoptosis, Pediocin PA-1 is also believed to interoperate with TLRs in order to initiate cell death [79].

*Phenazine*

Phenazine displays another group of bacteriocin, nitrogen containing metabolites, such as phenazine 1-carboxylic acid and phenazine 1,6-di-carboxylic acid (PDC) that are secreted by many bacterial strains, including a remarkable number of *Pseudomonas*. Phenazines are crucial for biofilm synthesis and help to protect bacteria from competitive microorganisms because of their toxicity [80]. In previous studies, PDC secreted from *Pseudomonas aeruginosa* was demonstrated to show substantial cytotoxic activity against CRC cells (HT29) among other cancer cell lines (HeLa, MCF7, DU145) in a dose-dependent manner. Thereby, its range of cytotoxic action was the greatest against colorectal HT29, HeLa and MCF7 [80,81]. In addition, PDC was observed to negatively affect both cancer cell viability and DNA synthesis and to induce G1 cell cycle arrest, thus initiating apoptosis [81].

*Azurin*

Azurin is another protein that is able to enter cancer cells and induce cell cycle arrest and apoptosis. The copper-containing protein found in *Pseudomonas aeruginosa* and its peptide p28 have even been studied in clinical trials of phase 1 already that demonstrated p28’s anti-cancer toxicity and safety [82,83]. As a multi-targeting peptide, p28’s anti-cancer activity is based on several mechanisms such as complex formation with p53 tumor suppressor, interferential action on the receptor of tyrosine kinase EphB2-mediated signaling process, reduced activity of VEGFR-2 tyrosine kinase, prevention of angiogenesis and interferential activity on P-cadherin protein expression [26,84]. These findings were also supported by another study, where azurin was demonstrated to decrease CRC cell viability by apoptosis induction, whereas non-cancer cells remained unaffected. This finding further underlines the potential of azurin as a selective anti-cancer agent [85].

*Exotoxin A*

Exotoxin A (PE) is also derived from *Pseudomonas aeruginosa* and represents the most toxic virulence factor of this bacterium [86]. The lethality of PE is based on its adenosine diphosphate (ADP)–ribosylation activity, leading to inactivation of the eukaryotic elongation factor 2 (EF-2) and thus inhibition of host cells protein biosynthesis [86,87]. These toxic properties have been observed to act as useful anti-cancer agents, since the active domain of PE has been found to specifically target tumor-related antigens [86]. Based on this background, Shinohara et al. fusioned a mutated PE with the variable regions of a monoclonal antibody directed against the human transferrin receptor to obtain a single-chain immunotoxin, namely HB21(Fv)PE40, in order to analyze its efficacy against murine metastatic CRC cells (KM12L4), and could demonstrate them to be eliminated when systemic administration of HB21(Fv)PE40 was applied [88]. In another study, Shapira et al. established another immunotoxin, namely SWA11-ZZ-PE38, which was created by conjugating SWA11 with a modified derivate of PE (PE38) via an Fc-binding ZZ domain from staphylococcal protein A to determine its efficacy against human CRC cells (HCT116, HT-29, COLO320). The in vivo study revealed that SWA11-ZZ-PE38 is able to induce apoptosis in human HT-29, COLO320 and HCT116 cell lines without being cytotoxic in normal tissue. These results seem to be another promising treatment approach for CRC by selectively and effectively targeting CRC cells without causing damage to vital tissue [89].

*Listeriolysin O*

Listeriolysin O (LLO) is a toxin produced by the facultative anaerobic bacteria *Listeria monocytogenes* that can enter the cytoplasm of antigen presenting cells, because of LLO’s hemolytic activity, penetrating the phagosomal membrane. In phase I and II of clinical trials, *L. monocytogens* have been widely utilized as a vaccine vector to stimulate immune responses to fight human cancer [90]. For example, Lm-LLO-E7, a recombinational form of *L. monocytogenes* (rLm) producing the human papilloma virus-16 E7 protein that is expressed in cervical cancer cells fusioned with LLO, has been demonstrated as being capable of inducing a potent anti-tumor response [91]. In another study, LLO from *L. monocytogenes* was fusioned to a HER2/neu oncogene expressing protein (ADXS31-142) that has been shown to exhibit anti-tumor effects in a variety of human carcinomas, including the Colo205 CRC cell line [92].

**Table 1:** Collection of bacterial peptides proposed for CRC therapy.

Protein/Peptides	Bacterial Source	Mode of Action	Refs.
**Enterotoxin (CPE)**	*Clostridium* *perfringens*	Binds to Claudin-3/-4 surface receptors and leads to CRC cell lysis in SW480, HCT-116, SW620, Caco-2, HT-29 and PDX CRC cells.	[62,63]
		Induces TNF-α-upregulation leading to decreased Claudin-4 expression, disrupted tight junctions, reduced EMT-, adherence- and metastasis-capacity in HT-29 CRC cells.	[93]
**Diphteria toxin (DT)**	*Corynebacterium diphteria*	Subunit A blocks protein synthesis by ADP-ribosylation of EF-2 leading to cell death.	[65,66]
		Non-toxic CRM197 suppresses angiogenesis and proliferation in SW480, SW620, HCT-116, Caco-2 and HT-29 CRC cells.	[67,68]
**Nisin**	*Lactococcus lactis*	Leads to apoptosis via promoting hole formation in the cell membrane of Caco-2 and HT-29 CRC cells.	[69]
		Prevents expression of MMP-2, MMP-9, CDTs and Cif in SW480, HT-29, Caco-2 and LS-180 CRC cells.	[70,71]
		Increases cytotoxicity of anti-cancer agents resulting in lower doses necessary for treatment.	[70]
**Colicin**	*Escherichia* *coli*	Acts cytotoxically through membrane hole formation, and non-specified DN/RNase activity in HT-29 CRC cells.	[72,73]
		Subunits A, E1, E3, U inhibit cell growth and promote apoptosis in HT-29 CRC cells.	[73,74]
**Microcin**	*Klebsiella pneumonia*	Pore forming into cell membranes, thus leading to apoptosis in CRC cells and other cancer cell lines such as HeLa.	[75,76]
		Subunit E492 shows a noticeable cytotoxicity especially in HT-29 but also in SW620 CRC cells.	[94]
		Subunit E492 reduces tumor proliferation in a xenograft model with SW620 CRC cells.	[94]
**Pediocin**	*Pediococcus acidilactici*	Shows lethal effects on HT-29 and DLD-1 CRC cells.	[78]
		Subunit PA-1 interoperates with TLRs and initiates cell death.	[79]
		Inhibits cancer cell proliferation as a carrier combination.	[95]
**Phenazine**	*Pseudomonas aeruginosa*	Subtype PDC weakens viability and DNA synthesis and initiates cell cycle arrest leading to apoptosis in HT-29 CRC cells.	[80,81]
**Azurin**	*Pseudomonas aeruginosa*	Influences p53/EphB2/VEGFR-2 signaling pathway and prevents angiogenesis in CRC cells.	[26,84]
		Inhibits cancer cell mobility and shows strong anti-cancer effect in HCT-116 CRC cells.	[96]
**Exotoxin A (PE)**	*Pseudomonas aeruginosa*	Inhibits EF-2 and protein biosynthesis via ADP-ribosylation, induces apoptosis in HCT116, HT-29 and COLO320 CRC cells.	[86,87,89]
		Subtype PE24-based amyloid injection leads to growth arrest and metastasis prevention in CRC-diseased mice.	[97]
**Listeriolysin O** **(LLO)**	*Listeria monocytogenes*	Acts as a membrane-damaging cytotoxin in Caco-2 CRC cells.	[98]
		Shows hemolytic activity and anti-tumor properties in Colo205 CRC cells.	[90,92]

*B: Non-Ribosomal Peptides*

Non-ribosomal peptides represent another class of peptides that can be synthesized by bacteria, fungi as well as cyanobacteria and represent another kind of metabolite displaying cancer protective properties, as well as against CRC, such as, for example, Lucentamycins, Arenamides, Ohmyungsamycins, Mixirins or Urukthapelstatin A, which are described in the following sections (Table 2) [52].

*Lucentamycin*

Lucentamycins (A-D)(3-methyl-4-ethylideneproline containing peptides) are isolated from the bacterial strain *Nocardiopsis lucentensis* and represent another type of peptide showing cytotoxicity against HCT-116 colon carcinoma cells [99]. However, not many studies investigating lucentamycin’s interactions with cancer, especially CRC cells, have been carried out yet.

*Arenamides*

In addition, new types of cyclohexadepsipeptides, namely Arenamides A-C, were found to be isolated from the marine bacterial species *Arenamides Salinispora arenicola*. In previous research, Arenamides A and B have been demonstrated to block TNF-induced activation, thus suppressing the pro-inflammatory NF-κB signaling pathway as well as nitric oxide and prostaglandin E2 production. Moreover, cytotoxic activity against human colon carcinoma cells (HCT-116) was observed [100].

*Ohmyungsamycin*

Other bioactive metabolites showing cancer cytotoxic action are the cyclic depsipeptides Ohmyungsamycin A and B that are derived from a marine bacterial strain (SNJ042) belonging to *Streptomyces* sp. and are both comprising unusual amino acid units such as N,N-dimethylvaline, β-hydroxyphenylalanine and N-methyl-4-methoxytrytophan [101]. In particular, Ohmyungsamycin A has been demonstrated to exhibit growth inhibiting effects on CRC cells (HCT-116), while it did not affect the growth of fibroblast cells (MRC-5), suggesting that it is able to selectively suppress the proliferation of human cancer cells [102]. Thereby the underlying mechanisms leading to its anti-cancer activity in CRC cells, namely modulation of the Skp-p27 axis leading to MCM4-induced cell cycle arrest in G0/G1 phase, finally causing apoptosis, have been revealed [102].

*Mixirins*

Mixirin is a cyclic acylpeptide that is derived from the marine *Bacillus* species. All three types of mixirins, namely A, B and C, have been found to exhibit cytotoxicity against the HCT-116 human colon tumor cell line, but further studies on mixirin, specifically considering CRC, have not been carried out yet [103].

*Urukthapelstatin*

The cyclic thiopeptide Urukthapelstatin A is secreted by *Mechercharimyces asporophorigenens*, a marine strain that belongs to the *Thermoactinomycetaceae*. Previous studies have demonstrated the anti-cancer activity of Urukthapelstatin A as they found growth inhibition colon cancer (HCT-116) cells among other types of cancer, when treated with Urukthapelstatin A, probably due to inhibition of DNA synthesis [104,105]. However, further studies need to be made for validation.

**Table 2:** Collection of non-ribosomal peptides proposed for CRC therapy.

Protein/Peptides	Bacterial Source	Mode of Action	Refs.
**Lucentamycin**	*Nocardiopsis lucentensis*	Shows cytotoxicity and induces apoptosis against HCT-116 CRC cells.	[99]
**Arenamides**	*Salinispora arenicola*	Subtypes A and B block TNF, nitric oxide and prostaglandin E2 and act cytotoxic on HCT-116 CRC cells. Investigation for chemopreventive and anti-inflammatory properties in HCT-116 CRC cells is proposed.	[100]
**Ohmyungsamycins**	*Streptomyces* sp.	Subtype A modulates Skp-p27 axis leading to cell cycle arrest (G0/G1 phase), apoptosis and selectively targeted reduction of proliferation in HCT-116 CRC cells.	[99,102]
		Subtype A shows stronger activity against human cancer cells compared to subtype B.	[106]
**Mixirins**	*Marine Bacillus* sp.	Subtypes A, B and C act cytotoxic against cancer cells and inhibit cell growth in HCT-116 CRC cells.	[103]
**Urukthapelstatin**	*Mechercharimyces asporophorigenens*	Subtype A inhibits DNA synthesis, growth and proliferation of HCT-116 CRC cells.	[104]

Altogether, the efficacy of bacterial toxins as well as non-ribosomal peptides, acting as cytotoxic agents in order to suppress cell proliferation, offer a great opportunity as selective anti-tumor agents because they can interfere with different cancer-promoting signaling pathways. Moreover, the cancer-inhibiting mechanisms have already been shown across different CRC cell lines in vitro and already in a few animal models (Table 1 and Table 2). However, these studies should be extended to gain further insights and to initiate clinical trials in the future.

#### 2.1.3. Bacteria as Carriers for Therapeutic Agents

Besides using bacteria and their metabolites as directly targeting cytotoxic anti-cancer agents, they have also been demonstrated to be used as therapeutic carriers in the treatment of CRC. Several studies have shown that bacterial carriers are not only able to selectively target cancer cells but also metastases [31,107]. Using imaging techniques such as bioiluminescence has been exhibited as a non-invasive method making it possible to detect and monitor tumors, including CRC, and even metastases by generating bioiluminescent cancer-colonizing bacteria offering novel opportunities in cancer diagnosis and treatment [29,31]. Moreover, using *Lysteria monocytogenes* as a vector for an anti-cancer vaccine has been shown to promote a remarkable amplification of its anti-cancer effects, whereby the intracellular microorganism is able to pass intestinal membranes to then trigger immune responses by activating CD8^+^ and CD4^+^ T-cells. In addition, the use of bacterial vectors can be considered as a safe treatment method due to clinical trials [27,28]. Another bacterial microorganism used as a carrier in the treatment of CRC is represented by the species of *Clostridium novyi*-NT, a non-toxic variant of the superior cancer-colonizing strain *Clostridium novyi*. The application of *C. novyi*-NT spores to tumor mouse models has even been shown to sprout within avascular regions and thus eradicate CRC cells. In combination with conventional chemotherapy, *C. novyi*-NT administration even exhibited hemorrhagic necrosis within 24 h after application, demonstrating a synergistic interaction against CRC cells and revealing *C. novyi*-NT as a promising bacterial carrier [30,108]. Therefore, using bacteria as therapeutic carriers in order to detect, target and fight viable cancer cells and metastases represents a powerful tool in the treatment of CRC.

#### 2.1.4. Bacteria-Mediated Anti-Angiogenesis Therapy

Another treatment strategy using bacteria is demonstrated by anti-angiogenesis therapy, going hand in hand with tumor growth suppression. The formation of new blood vessels, so called angiogenesis, is crucial for solid tumors to grow and metastasize. Therefore, blocking angiogenesis represents another promising target in cancer treatment [40,109]. In previous research, a genetically attenuated bacteria strain of *Salmonella* sp. (VNP20009) was used to administer as a combination therapy together with angiogenesis inhibitor rhEndostatin in tumor mice models with the aim of targeting angiogenesis, thus tumor growth and proliferation [110]. Since separate application of only bacteria or only rhEndostatin have shown little impact on tumor proliferation, the combination of both exhibited great effects in targeting the tumors and suppressing their growth, indicating unique metabolic properties of bacteria that help to complement or even enhance anti-angiogenesis effects of systematically administered agents in cancer therapy [110]. In other studies, bacterial strains, namely *Bifidobacterium adolescentis* [111] and *Bifidobacterium longum* [112], were used as Endostatin vectors for targeting tumor angiogenesis, showing significant results in tumor growth inhibition [111,112]. Moreover, Niethammer et al. have found evidence that an oral anti-angiogenic bacterial DNA vaccine, carried by attenuated bacterial strain *Salmonella typhimurium* and directed against VEGFR-2, displayed remarkable anti-cancer effects in different tumor types, including CRC [113].

#### 2.1.5. Bacterial Biofilms

Biofilm is an ancient type of multicellular life, more precisely it describes a community of microbes that is either attached to a surface or encompassed in an extracellular matrix, giving bacteria resistance to drugs and hosts defense mechanisms [114,115]. Biofilms that are, for example, found in bacterial pathogens such as *Salmonella tyhimurium*, *Pseudomonas aeruginosa* or *Staphylococcus aureus* are responsible for causing many chronic diseases and thus play an important role in their pathogenesis [116,117]. *Salmonella tyhimurium* and other infections have been linked to significant tumor hemorrhage. When hemorrhage is activated, T-cell production is induced as an important feature for the formation of biofilms [116]. Besides the pathogenic role of biofilms in a wide range of diseases [116,117], their potential as a novel treatment strategy in cancer therapy has just been discovered [118]. Moreover, it has been reported that metastasis can be prevented through bacterial biofilm formation burying cancer cells during the SOS response [118]. These findings indicate bacterial biofilms being able to influence CRC formation and progression by altering cancer metabolome and regulating cellular proliferation, to have great potential as an effective anti-cancer agent in CRC treatment [118]. On top of that, previous research found evidence that the bacterial macromolecules required for biofilm formation (proteins and DNA) are helping to block cancer cells to metastasize by simply coating them [119]. Regarding metastasis, polysaccharides produced by *Streptococcus agalactiae* have been revealed to prevent cancer cells from adhering to endothelial cells, thus blocking an essential stage of metastasis and disease progression [120]. However, the opportunities to use bacteria biofilm in the treatment of CRC, especially for metastasis distraction, need to be further investigated.

#### 2.1.6. Maintaining Microbial Equilibrium

When the microbial equilibrium is disrupted, pathogenic microorganisms may be prevalent in the gut, potentially leading to the pathogenesis of CRC. Therefore, altering the bacterial composition and reestablishing, if disrupted, the critical balance between different bacteria species could represent a new therapeutic approach helping to treat CRC [34]. The role of microbiota in the development of CRC has increasingly emerged since several studies found significant differences in the composition of the intestinal microbiome between CRC patients and healthy individuals, whereas specific microbes and bacterial strains that are enriched in CRC pathogenesis could be, at least in part, identified [121,122]. In general, CRC patients often show a reduced microbial diversity compared to healthy individuals, whereas bacterial strains including *Streptococcus bovis*, *Helicobacter pylori*, *Bacteroides fragilis*, *Enterococcus faecalis*, *Clostridium septicum*, *Fusobacterium* spp. and *Escherichia coli* are suspected to play a critical role in carcinogenesis of CRC [121,123,124,125]. It is assumed that these specific bacteria trigger CRC by different mechanisms, such as increased release of bacterial toxins and pro-carcinogenic compounds leading to mutagenesis, reduced bacterial synthesis of health-beneficial metabolites, destruction of the epithelial barrier and microbiotic alterations and dysbiosis, which finally promote abnormal immune activity leading to chronic inflammation, stimulating CRC development and progression [123,126,127]. Although it is still not fully understood if the disruption of microbial balance and dysbiosis acts as a cause or consequence of CRC tumorigenesis and which factors interact with each other and contribute to CRC development, the model of bacteria-induced CRC mechanisms has greatly contributed to finding new options in CRC therapy. These approaches include the supplementation of inflammation-protective microbial fermentation products, elimination and suppression of bacteria-released toxins with inhibitor molecules, enhancing anti-tumor treatment efficiency by using specific bacteria such as Bifidobacterium, as well as consumption of probiotica [123]. Probiotics have the ability to direct cells into a vital state by modulating the immune system, lowering blood cholesterol and decreasing colitis [35], whereby each probiotic has a distinct function that could be beneficial for CRC prevention [35]. For example, probiotics have been shown to play a pivotal role in reducing pro-inflammatory cyclooxygenase-2 expression, which is involved in tumor angiogenesis, hence contributing to carcinogenesis suppression [128]. In addition, anti-inflammatory properties of probiotics were also shown by the down-regulation of the master regulator of inflammation NF-κB and its associated signaling pathways. This further highlights the major potential of probiotics in reducing inflammation through NF-κB modulation and thus preventing inflammation-associated diseases such as CRC [44,45,128,129]. Moreover, Sivan et al. showed the great impact of probiotics on increasing the anti-cancer activity of anti-PDL1 medicine [130], demonstrating that intestinal bacteria can remarkably affect both immunotherapy and chemotherapy in order to promote anti-cancer effects. Moreover, *Lactobacillus acidophilus* represents another probiotic species that has been shown to reduce the occurrence of colorectal tumors as well as the size of tumors in mice, whereby the bacteria has been discovered to have anti-cancer effects through enhancing serum levels of IFN-, IL-10 as well as the number of CD4^+^ and CD8^+^ cells, while drastically lowering serum levels of CEA and CA19-9 tumor markers [33]. In addition, *Lactobacillus brevis* SBL8803, which has been identified in fermented malt, was also shown to exhibit anti-colon cancer properties. Hereby, polyphosphate actin as an anti-cancer chemical is produced by *L. brevis* 8803 that has been demonstrated to promote apoptosis in SW620 cells by activating the ERK pathway, whereby it has been proposed to act as a less toxic anti-tumor agent compared to standard cancer medicines [131]. Another species of *Lactobacillus*, showing anti-cancer properties is represented by *Lactobacillus casei* BL23. In a study model of colon cancer triggered by azoxymethane and dextran sodium sulfate, *Lactobacillus casei* was able to modify the immune response and thereby to reduce adenoma formation. Together the study’s findings revealed that *L. casei* BL23 was able to protect mice from CRC by suppressing tumor formation and proliferation as well as showing a great immunomodulatory impact, highlighted by the downregulation of IL-22 and overexpression of Caspase-7, Caspase-9 and Bik [132]. Besides *Lactobacillus, Bifidobacteria* as another probiotic family, in particular *Bifidobacterium longum, Bifidobacterium infantis, Bifidobacterium adolescentis* and *Bifidobacterium breve* have been recognized to act as CRC suppressors, since they are able to fight CRC by modulating the immune response, binding and degrading potential carcinogens as well as maintaining a healthy balance in the intestinal microflora, producing anti-tumorigenic or anti-mutagenic agents in the colon and altering metabolic activities of the intestinal microflora [133,134]. For instance, in previous research, it has been shown that CRC cell lines such as Caco-2, HT-29 and SW480 were inhibited by butanol extract from *B. adolescentis* SPM0212. Furthermore, *B. adolescentis* SPM0212 was demonstrated to activate macrophages and dramatically enhance the production of TNF and nitric oxide, boosting the immune response activity to control immunological modulation and tumor cell death [135]. Overall, on the one hand probiotics have been shown to help prevent CRC development and to keep the intestinal micro-ecology in balance. On the other hand, they have been shown to be effective as CRC proliferation suppressors and anti-cancer immune modulators.

## 3. Microbiota in CRC

### 3.1. Influence of Microbiota on Drug Metabolism

With a bacteria-to-cell ratio of roughly 1:1 in the human body, microbes encode for 150 times more genes than the human genome [19]. The discovery of microbiota-specific metabolic signatures contributes to a better knowledge of the relation between bacteria and human cells and several studies have demonstrated that microbiota-dependent metabolites have a great impact on the immune function, therefore better understanding could aid in the prediction of drug effects and outcomes in their application.

Han and colleagues used a library of 833 metabolites to describe the metabolic identities of 178 gut bacteria with mass spectrometry and a machine learning workflow by using murine serum, urine, feces and caecal contents [136,137]. In this study, they could precisely map genes according to bacteria’s metabolism and their phenotypic variation as well as associate metabolites with microbial strains. For example, *Firmicutes* and *Actinobacteria*, which are two phylogenetically distant strains were found to produce high levels of ornithine, which is important for the regulation of several metabolic processes, whereas *Enterococcus faecalis* and *Enterococcus faecium* were demonstrated to accumulate high levels of tyramine that is known to modulate neurological functions. On the other hand, *C. cadaveris* has been shown to act as a consumer instead of a producer and to consume high levels of vitamin B5 that is linked to inflammatory bowel diseases [136,138,139].

These observations highlight the great potential of better knowledge about microbiota-dependent metabolites in drug therapy, because orally delivered chemicals are mainly absorbed in the gut and therefore represents the site where the majority of metabolic changes of medication takes place [137].

Because medications have a significant impact on microbiota composition and balance, it is critical to bring up the interacting relationship between drug components and the microbiome [140]. Anti-diabetics, proton pump inhibitors [140] and nonsteroidal anti-inflammatory medications are all representations of drug-induced toxicity on microorganisms [33]. However, bacteria have also been discovered to have the ability to digest medicines. In a previous study, Maier et al. applied 1197 medicines from various therapeutic classes to 40 distinct bacteria species, excluding antibiotics, in an attempt to widely and thoroughly address these effects [140]. The researchers found that almost 30% of the substances examined hindered the proliferation of at least one bacterial species, therefore they hypothesized that antibiotic resistance may also arise as a result of changes in the microbiota caused by non-antibiotic exposure [140]. Genetic screens, and enzymatic analysis to find enzymes promoting specific drug conversions, have been used to investigate the reasons and effects of drug-microbiota interactions [141]. Recently, the metabolism of gut microbiota has gained more attention since it may explain why individuals suffering from the same disease and undergo the same treatment, show different therapeutical outcomes. Moreover, it shows the complex and challenging task to find an efficient treatment strategy for every individual. In order to find appropriate drugs for every patient, machine-learning frameworks using network-based analyses and data to identify drug biomarkers predicting drug responses increasingly take place [142]. With machine learning models and artificial intelligence, individual-specific cancer therapy can be developed to help improve therapeutic outcomes [142,143]. Furthermore, identifying hazardous by-products of bacterial medication aids in the prediction of potential adverse effects in patients undergoing therapy. With the wide spectrum of impacts of bacteria-induced chemical metabolism, such as pharmacological activation [144], inactivation [145] or toxicity [141], pinpointing the bacteria or their characteristics causing a specific metabolic effect is currently one of the most challenging aspects of treatments. For example by influencing the TNF response or ROS production [146], metabolic processes of glucuronidation conjugating pharmaceuticals to glucuronic acid (GlcA) in the liver, inactivates and detoxifies medicines. These glucuronides are then taken to the gut and are eliminated from the body [147]. However, once in the colon, these compounds can be reactivated by gut bacterialglucuronidases (GUS) enzymes by removing the GlcA, resulting in local acute toxicity [148]. Furthermore, as customized medicine is becoming increasingly important, research is currently being conducted into the extent to which individual drug metabolism can be harnessed. Javdan et al. created a technique to find metabolites formed by microbiome-derived metabolism (MDM) enzymes in a series of 23 orally applied medicines in human healthy donors in order to describe metabolic interactions between microbiota and therapeutical agents [149]. This study included different methodologies, including microbial community cultures, small-molecule structural assay, quantitative metabolomics, metagenomics, mouse colonization and bioinformatic analysis, making it a very extensive and technically heavy approach. The authors demonstrated the efficacy of this technique in identifying MDM enzymes in a high throughput manner utilizing medicines from several groups with varying mechanisms of action [149]. Zimmermann et al. used a related attempt to assess the in vitro ability of 76 naturally occurring bacteria in the human gut to metabolize 271 orally administered pharmaceuticals from various groups based on their mode of action. Surprisingly, at least one of the microbes studied was shown to metabolize up to two-thirds of the medications tested [150]. Furthermore, a single microbe had the ability to digest up to 95 distinct medicines and they were able to discover distinct drug-metabolizing gene products that are accounting for the conversion of medicines into metabolites using metabolomics, mass spectrometry and DNA sequence analysis [150]. Finally, in silico techniques have been created to enable the characterization of pharmaceuticals and their metabolites by certain bacterium species [140] as well as the prediction of toxicity events using data on bacteria composition, drug activity and food preferences [151]. When it comes to medication metabolism in the human body, more evidence has pointing out the importance of gut microbiota, as bacteria and their metabolites can affect pharmacokinetics and pharmacodynamics, which is a significant finding in context to therapy. In the next chapter, we will focus on how the microbiome affects traditional CRC therapy.

### 3.2. Influence of Microbiota on Conventional CRC Therapy

In conventional CRC therapy, chemotherapeutic agents and radiation are used and, due to their insufficiency, co-treatment with supplements, phytopharmaceuticals or feces transplantation, with its influence on the microbiome, are becoming increasingly interesting. Chemotherapeutics have been utilized for decades to treat a variety of human tumors and still represent typical first-line treatment for CRC [152], but are also used in combination with fluoropyrimidine-based substances and oxaliplatin as well as irinotecan [153] at the advanced, non-resectable CRC stage. Nonetheless, a substantial number of patients are likely to experience treatment-related morbidity and mortality due to these medications [152]. Given that CRC develops in close neighborhood to gut bacteria, new research has focused on how the gut microbiota influences the efficacy and toxicity of existing chemotherapeutic treatments [146]. Traditional CRC medicines such as irinotecan, 5-FU and cyclophosphamide have been demonstrated to alter the microbiome diversity of mice in pre-clinical models as well as in human patients. However, it is still unclear how this affects the prognosis, as some research revealed conflicting results when it comes to the role of microbiota in therapy. For example, in an animal experiment, germ-free mice were much more resistant [154] to powerful anti-cancer agent irinotecan [155] and had a higher lethal dose than holoxenic mice [154]. This could be due to the development of metabolites that are harmful to drugs as a consequence of bacterial metabolism. The authors have not thoroughly investigated the ultimate cause of death of these mice and did not identify the crucial bacterial species that accounted for this phenomenon. However, interestingly, irinotecan’s major side effect of diarrhea correlating with intestinal damage was very rarely observed in germ-free mice compared to holoxenic animals [154], while irinotecan-treated patients often show severe diarrhea as a side effect. In their liver, irinotecan is converted to its active form, human topoisomerase I poison SN-38, and then inhibited by DP-glucuronosyltransferases by adding GlcA (SN-38-G) [156]. This inactive compound is revived by GUS in the colon, resulting in acute poisoning. Jariwala et al. discovered the GUS enzymes responsible for SN-38 reactivation in the human gut using a combination of proteomics and bioinformatic analysis on human feces samples under the consideration that SN-38 is a harmful metabolite of irinotecan [148]. Meanwhile, it is known that removing GlcA from SN38-G causes SN38 reactivation, leading to the described disadvantages for the patients. Inhibition of the GUS enzyme synthesis thereby minimizes intestinal damage and maintains irinotecan’s anti-cancer activity [156]. These findings imply that the presence of some bacteria is responsible for an increase in treatment-associated adverse effects leading to the assumption that gut microbiome can influence therapeutic efficacy. Surprisingly, bacteria appear to have a dual function in cancer treatment, with studies reporting a synergistic impact of microbiota and therapeutic efficacy, while some others demonstrate the presence of bacteria as an barrier for the efficacy of drug [153]. With regard to diseases of the digestive organs, research is constantly being conducted into the potential effects of nutritional supplements. More than a decade ago, it was shown that supplementing a high-inulin or oligofructose diet inhibited the growth of a transplantable tumor in a mouse model. Inulin and oligofructose are fructans that have been found to increase Bifidobacteria proliferation in the stomach. The inclusion of these supplements to the animals’ food increased the efficacy of six different chemotherapy medicines, namely 5-FU, doxorubicine, vincristine, cyclophosphamide, methotrexate as well as cytarabine, implying a prebiotic impact of inulin and oligofructose [157]. An auspicious approach is offered by phytopharmaceuticals, safe secondary plant compounds with numerous health-promoting effects ranging from anti-inflammation to tumor containment. The treatment of CRC cells with resveratrol [7,158,159] or the components of *Curcuma longa* (turmeric) curcumin [160] and calebin A [13,161,162,163] is particularly promising, as these substances can extensively modulate tumor processes. In in vivo-like models, it was shown that all of the three phytopharmaceuticals mentioned above enhance the effect of the cytostatic drug 5-FU [163], and since they alter not only the CRC cells but also the immediate environment as part of their anti-tumor effect, it is obvious that they can also have an influence on the intestinal microbiome.

Another interesting approach is fecal microbiota transplantation (FMT), firstly introduced in 1958 for treatment of *Clostridium difficile* infection (CDI) [164]. Here, up to 80% of all CDI cases could be treated by assisting in the restoration of a beneficial microbiome in infected patients. In addition, FMT was found to be successful in a variety of other illnesses, including inflammatory bowel diseases, diabetes or even autism, thus it became a viable therapy option [165]. The benefits of this method were also addressed as a way to mitigate undesirable effects from radiation treatment due to its safety. For CRC treatment, radiation is utilized as a standard therapeutic strategy in conjunction with chemotherapy [6], where patients may have a variety of severe adverse effects, such as bone marrow and gastrointestinal damage, thus bacteria have been shown to reduce these adverse effects of radiation treatment in pre-clinical trials and, furthermore, in various pre-clinical cancer mouse models, the gut microbiota has been found to influence even the efficacy of radiation [166,167]. Furthermore, worth mentioning, it was shown that applying certain bacteria such as *Lactobacillus rhamnosus* to mice undergoing radiotherapy had a protective impact on the intestinal mucosa of the tested animals [168]. Moreover, probiotics were found to reduce radiation-induced gastrointestinal damage in cancer patients undergoing irradiation in clinical investigations such as diarrhea [150].

The future of cancer therapy will undoubtedly lie in the investigation of the dual function of microbiotica in medication outcomes: on the one hand, though bacteria is able to exacerbate therapy side effects as a result of their metabolism, on the other hand the existence of microorganisms is critical for the efficacy of cancer therapeutical agents [63,166], playing a special role in CRC and its treatment because of the bacteria-rich digestive organs.

## 4. Discussion and Perspectives

The use of bacteria in cancer therapy is often overlooked, although there is great evidence that this kind of treatment represents a promising chance to cure patients. In fact, bacteria and their different compounds can act as a double-edged sword when it comes to CRC, since specific species have been demonstrated as cancer-stimulating and triggering agents, whereby other bacterial strains show highly selective anti-cancer properties without cytotoxicity to vital tissue.

Moreover, many of these bacterial agents have only been investigated in pre-clinical trials and detailed information about their toxins and metabolites is still limited, thus further research on their mode of action and properties in general is needed. In addition, the risk of uncontrollable complications of therapeutic bacteriotherapy due to infections represents another limitation for bacteriotherapy to reach full acceptance in CRC treatment. However, several attempts have been made to overcome these issues, for example, by attenuating or even eradicating toxic compounds of bacteria as well as approaches to modify specific strains to reduce the risk in treatment application. Besides using bacterial agents or their toxins themselves in CRC therapy, specific bacteria have also been demonstrated to act as potential drug carriers due to their unique chemical characteristics such as low molecular weight and hydrophobicity together with their tumor- and metastasis targeting properties, while showing less side effects than conventional cancer treatment. Additionally, bacteria are not directly coupled to the specific extracellular or intracellular receptors so that bacterial anti-cancer peptides are able to prevent various resistance processes [169]. As another downside of bacteriotherapy the short half-life of bacterial peptides has to be mentioned, displaying a major problem in the application. However, their chemical modifications have already been worked on, such as the substitution of D-amino acids, cyclization or the replacement of labile amino acids among other methods to improve the half-life and stability to make them more efficient for therapy [170,171,172].

Altogether, it is of great importance to understand that bacteria and bacterial agents in application as well as the composition of our microbiome is a matter of great complexity and can hardly be considered separately. Therefore, better understanding of bacterial interactions, their metabolism in context of drug administration as well as effects of our microbiome are necessary and further use of artificial intelligence and machine learning is needed to develop customized high-efficiency therapy for individuals suffering from CRC.

## 5. Conclusions

Overall, bacteria as a novel treatment strategy in CRC and are of major potential in many aspects, although bacteriotherapy alone may not exert all the therapeutic extent. Therefore, using bacteriotherapy in the form of preventive, concomitant or as a kind of anti-cancer agent carrier therapy as well as utilizing the individual microbiome to develop the most efficient therapy for every individual might help to exploit the full potential of bacteria-mediated therapy in the fight against CRC. However, more clinical trials and in vivo studies are necessary as well as further identification of microbiota-specific features to establish bacteriotherapy as a prestigious strategy in CRC treatment, whereas the complexity of bacteria and the microbiome together with associated interactions in therapeutic applications has to be further discussed as a whole in the future.

## Data Availability

All data are available in the manuscript.

## Abbreviations

5-FU	5-fluorouracil
Bad	Bcl-2-Antagonist of Cell Death
Bak	BCL2 Antagonist/Killer 1
Bax	Bcl-2-associated X protein
Bcl-1	B-cell lymphoma 1
CD	cluster of differentiation
CDI	*Clostridium difficile* infection
CDT	cytolethal distending toxin
Cif	cycle inhibiting factor
CPE	*C. perfringens* enterotoxin
CRC	colorectal cancer
DT	diphteria toxin
EF	elongation factor
FMT	fecal microbiota transplantation
GlcA	glucuronic acid
GUS	gut bacterial-glucuronidases
IFN	interferon
IL	interleukin
LLO	Listeriolysin O
MDM	microbiome-derived metabolism
MMP	matrix metalloproteinase
NF-κB	nuclear factor ‘kappa-light-chain-enhancer’ of activated B-cells
PCA	phenazine 1-carboxylic acid
PDC	phenazine 1,6-di-carboxylic acid
PE	Pseudomonas aeruginosa endotoxin
TNF	tumor necrosis factor
TNF-R	Tumor necrosis factor receptor
TLR	Toll-like receptor
VEGF	Rvesicular endothelial growth factor receptor
